# Benefits of the Light Consumption of Red Wine in Pain, Tender Points, and Anxiety in Women with Fibromyalgia: A Pilot Study

**DOI:** 10.3390/nu15153469

**Published:** 2023-08-05

**Authors:** María Victoria González-López-Arza, José Vicente Triviño-Palomo, Jesús Montanero-Fernández, Elisa María Garrido-Ardila, Blanca González-Sánchez, María Jiménez-Palomares, Juan Rodríguez-Mansilla

**Affiliations:** 1 ADOLOR Research Group, Department of Medical-Surgical Therapy, Faculty of Medicine and Health Sciences, Extremadura University, 06006 Badajoz, Spain; mvglez@unex.es (M.V.G.-L.-A.); jvtrivio@yahoo.es (J.V.T.-P.); blgonzalezs@unex.es (B.G.-S.); mariajp@unex.es (M.J.-P.); jrodman@unex.es (J.R.-M.); 2 Mathematics Department, Faculty of Medicine and Health Sciences, Extremadura University, 06006 Badajoz, Spain; jmf@unex.es

**Keywords:** fibromyalgia, wine, pain, anxiety, fibromyalgia impact questionnaire

## Abstract

Background: Fibromyalgia (FM) is characterized by chronic widespread pain, as well as anxiety, sadness, and depression. These symptoms are present in most patients and have a negative impact on their daily, family, and social life. The role of neurotransmitters in the pathophysiology of FM has been extensively discussed. The scientific evidence shows that levels of serotonin are decreased in patients with FM. Numerous studies support the beneficial effects that moderate wine consumption has on the body, with cardiovascular, endocrine, bone, and muscle improvements. Objective: The objective of this pilot study was to assess whether light consumption of red wine improves the main symptoms of FM. Methods: The study consisted of an experimental study with a control group with a total of 60 women diagnosed with FM following the American College of Rheumatology’s criteria. The experimental group ingested 15 g of alcohol per day, in the form of red wine, over a period of four weeks. The outcome measures were: the level of pain in tender points, sadness, anxiety, depression, and quality of life. The assessments tools were: tender point graphics, the visual analogue scale (for the assessment of pain and sadness), the Hamilton Anxiety Scale, the Hamilton Depression Rating Scale, and the Fibromyalgia Impact Questionnaire. The measurements were completed before and after the consumption of red wine. In addition, the differences between groups were evaluated in terms of drug consumption in the pre-intervention and follow-up phases. Results: Statistically significant improvements were obtained in the wine ingestion group for the variables of pain (*p* = 0.038), tender points (*p* < 0.001), and anxiety (*p* = 0.028). An improvement in the mean values was observed in favor of the experimental group for the variables of sadness, depression, and quality of life. The differences observed in the changes seen in the groups that were in favor of the wine ingestion group should not be attributed to the consumption of drugs but to the fact that the experimental group had a light intake of red wine. Conclusions: The results of this pilot study suggest a potential relationship between alcohol intake through the light consumption of red wine as part of the patients’ diet and the improvement of the main symptoms of fibromyalgia. Future studies are necessary to confirm these preliminary data; a bigger sample and a controlled diet should be considered, and the mechanisms through which improvements are achieved should be analyzed.

## 1. Introduction

Fibromyalgia was recognized in 1993 by the World Health Organization as a rheumatologic illness [1]; it is a complex condition that is characterized by chronic and generalized pain [2,3]. The pain is often accompanied by other symptoms, such as anxiety and depression, which can have a negative impact on the patients’ undertaking of daily activities [4]. Furthermore, the social and employment-based repercussions of fibromyalgia are reaching epidemic proportions. Fibromyalgia affects, on average, 2.10% of the world’s population, 2.31% of the European population, and 2.40% of the Spanish population [5]. The prevalence of this disease is currently between 2% and 4%, with most cases in women (80–90% of the cases) who are between 40 and 49 years old [6]. In Spain, it represents 15% of rheumatologic consultations [7].

The cause of fibromyalgia is still unknown. The most accepted hypothesis is an exponential increase in pain sensitivity at both the peripheral and central neurological levels. In this sense, low levels of serotonin have been detected. This is an important substance for the regulation of pain and a neurotransmitter, one purpose of which is to regulate the intensity of how we perceive pain [4,8]. Fibromyalgia is a disease with a poor response to treatment; therefore, the scientific evidence is in favor of a multi-factorial approach to its management, including pharmacological and non-pharmacological treatments [4,7,9,10]

Wine is defined in Spain (by law 24/2003 on the 10 July) as “a natural foodstuff obtained exclusively by the total or partial process of alcoholic fermentation by using fresh grapes (squeezed or not) or grape must” [11]. Among the nearly 800 components of wine are ethanol, flavonoid polyphenols (such as resveratrol, a powerful antioxidant and one of the most thoroughly researched from a medical point of view), proteins, and amino acids (including tryptophan, an essential amino acid precursor to serotonin).

During the first International Congress on the Mediterranean Diet [12], wine was considered the most complete element of the diet and was included in the traditional food pyramid. The negative effects of wine are the result of excessive ingestion, which has been observed in both acute and chronic alcoholism [13,14]. Wine has been recognized to have antispasmodic and antibacterial properties, as well as an antihistamine effect [15,16]. It also triggers bile secretion, which causes stimulation in the olfactory and gustatory organs and produces minerals and trace elements [17]. Currently, there is a line of research orientated towards the study of the relationship between the responsible and moderate consumption of wine and its derived beneficial effects. It is known from medical evidence that moderate wine consumption exerts beneficial effects on health [18,19]. Moreover, it is widely recognized in the medical literature that patients with fibromyalgia have no effective treatment available for their numerous complaints, and are therefore polymedicalized, without results that improve their quality of life; this means that the approach to their symptoms must be multidisciplinary [20,21]. There are also many studies in the medical scientific literature that have evidenced the protective effect of wine on the cardiovascular system due to the effect that wine has on the endothelium [22,23]. It has previously been demonstrated in animals that the moderate consumption of wine produces anti-aging effects and inhibits the development of certain cancers and Alzheimer’s disease due to the antioxidant action of the polyphenols, which protect the membranes of neurons [24,25,26]. Some studies support the relationship between the moderate consumption of alcohol and an increase in bone mass in both men and women [27,28]. Medical research has shown that the consumption of ethanol leads to central and peripheral analgesic activity, as well as producing an anti-inflammatory effect [29,30]. In addition, the consumption of small quantities of wine can reduce inflammatory activity and pain in patients with rheumatoid arthritis [31] These effects can be extrapolated to the symptomatology of fibromyalgia, as it is also a rheumatic disorder. It has also been observed that red wine can increase blood serotonin levels [32,33]. The neurotransmitter serotonin, which is involved in the control of mood and emotion, has been found to be deficient in patients diagnosed with fibromyalgia. Therefore, the use of antidepressants targeting serotonin or noradrenaline function in the brain can only induce remission in one third of patients [34,35]. Hence, a larger percentage of people could benefit from the effects of moderate wine consumption.

On the other hand, red wine, which is rich in terms of its polyphenolic composition, including resveratrol (which has been shown to be useful in inhibiting the expression of PDE4, an enzyme influenced by the stress hormone corticosterone) and in tryptophan, triggers the release of endorphins, such as serotonin. This is why we believe that the light consumption of red wine, always under medical supervision, would be beneficial to fibromyalgia patients. [36,37,38].

Based on all this, the objective of the present study is to analyze whether the daily intake of 15 g of alcohol, in the form of red wine in the diet and under medical supervision, improves the main symptoms of women diagnosed with Fibromyalgia.

## 2. Materials and Methods

### 2.1. Study Design

We conducted a longitudinal pre–post-intervention pilot study of women diagnosed with fibromyalgia along with a control group. Ethical approval was received from the Bioethical commission of the University of Extremadura in Spain (registration number: 78/2007). All the participants gave their informed written consent, according to the mandate of the World Medical Association Declaration of Helsinki [39].

### 2.2. Participants

The target population was women diagnosed with fibromyalgia from the Fibromyalgia associations AFIBA (Badajoz, Spain) and AFIBOL (Olivenza, Badajoz, Spain). Considering that the intervention factor to be studied was an alcoholic beverage, we proposed a pilot study and were very strict in the selection criteria.

The inclusion criteria were as follows: women over 18 years of age who had been diagnosed with fibromyalgia following the American College of Rheumatology’s criteria. The exclusion criteria were: contraindications for the intake of alcohol under medical supervision, a family history of alcoholism, a personal intolerance for alcohol, and pregnancy.

In order to prioritize the principles of beneficence and non-maleficence, we decided not to carry out a randomized controlled trial but decided instead on a longitudinal before-and-after study with a control group. The intervention group consisted of the 30 women who met the inclusion criteria and the control group consisted of 30 women who also met the exclusion criteria. The allocation was conducted by a physician who was independent of the study.

The control group could not be blinded due to the characteristics of the study intervention. A placebo group was not used because this was a pilot study and the sample size was not sufficient for the participants to be divided into three groups.

In order to avoid any possible negative influences that seasonal changes might exert over the fibromyalgia patients [4,6,7], the study was carried out during 4 weeks in the month of June. For one week before the beginning of the study and during the whole intervention, the participants did not consume any type of alcoholic beverage apart from the wine intake established in the study design.

### 2.3. Data Collection and Outcome Measures

A structured questionnaire was used for the data collection. The study variables were age and the time since the fibromyalgia diagnosis, and the main outcome measures were the most frequent symptoms of fibromyalgia (pain, sadness, anxiety, and depression) and the patient’s quality of life. To collect data related to the diet, each participant was provided with a self-developed diary in which she had to record the solid and liquid foods eaten as well as the approximate amount eaten.

The consumption of medicines as potential contamination factors was recorded before and after the intervention (weeks 0 and 4) and during the intervention (weeks 1, 2, and 3). The intake of analgesics, anti-inflammatories, selective serotonin reuptake inhibitors (SSRIs), tricyclic anti-depressants, and benzodiazepines was recorded.

Two outcome measure assessments were performed: an initial evaluation at week 0 (pre-test) and another one at the end of the study at week 4 (post-test). The Fibromyalgia Tender Points Graph from the American College of Rheumatology was used for the assessment of the presence of points that were sensitive to touch [3]. All of the participants marked the points that felt painful above the corresponding points on the body chart, with a maximum of 18 points.

The Visual Analog Scale (VAS) was used to determine the pain and sadness scores. This is a test that has already been used by numerous authors as an evaluation tool for the symptomology of fibromyalgia patients. The patients marked in a horizontal VAS of 0–10 cm their perception of the variable [31]. These variables were also measured at weeks 1, 2, and 3.

For the evaluation of depression, we used the Hamilton Depression Rating Scale (HDRS) (reduced version), which has been validated in Spain by Ramos-Brieva [40,41]. It contains 17 items in the computerized version. In order to categorize the intensity of the disturbances caused by depression, we chose the following scale: 0–6 points = without depression, 7–17 points = light depression, 18–24 points = moderate depression, and 25–52 points = serious depression.

The Hamilton Anxiety Scale (HAS) was used for the assessment of the anxiety levels. We used a Spanish version developed by Carboles et al. [42] that constitutes a scale of 14 items. It ranges from 0 to 56 points and, if the score is above 15 points, the level of anxiety is deemed to be high.

In order to quantify the repercussions of fibromyalgia on the quality of patients’ lives, we used the Spanish version of the Fibromyalgia Impact Questionnaire (FIQ) validated by Rivera et al. [43]. If the score is greater than or equal to 70 (the scale ranges from 0–100), the patient is classified as “severely affected”.

All of the participants were shown how to fill out these questionnaires.

### 2.4. Interventions

The patients allocated to the experimental group had supervised wine intake in their diet, and those allocated to the control group did not receive any intervention. The study was conducted for 4 weeks.

The experimental group’s intake of wine was consumed twice a day: 70 mL with lunch and 70 mL with dinner. All of the wine used in the study and consumed by the participants was provided by the research team. A young red wine of the “Tempranillo” grape variety was chosen because the majority of the beneficial substances of wine (such as resveratrol) accumulate in the skin of this grape and are not lost in the wine-making process (during the aging of a wine in the barrel, a large quantity of non-flavonoid phenols are added to the wine, while the polyphenol flavonoids of the wine are lost). This wine originates from the Ribera del Guadiana region (Spain). It has a 13.5% alcohol concentration and a polyphenol content of 98.5% [44]. The characteristics of the wine are as follows: Ph 3.80, resveratrol 1.5 mg/L, volatile acidity 0.65 g/L, total acidity 5.4 g/L, sugars 3.10 g/L, malic acid 0.00, lactic acid 1.20 g/L, anthocyanin index 420, polyphenol index 98.50 g/L, free sulfur dioxide 20.00 mgr/L, and total sulfur dioxide 95 mgr/L. A glass with the milliliters to be consumed was marked and was provided to the participants of the experimental group in order to ensure they consumed the correct amount of wine. The daily quantity of wine was 15 g of alcohol, which is within the limits of safe consumption for women (<20 g per day) according to the WHO; it is also within the limits for the light consumption of alcohol (<110 g per week) according to international references [45], which corresponds to 140 mL of wine.

A total of 105 g of alcohol was drank per week. This quantity falls within the category of the light consumption of alcohol, according to international references [46], whereby the subjects were classified as moderate drinkers (110–280 g/week), light drinkers (<110 g/week), and non-drinkers. The control group did not drink any wine or any other type of alcohol for the duration of the study.

The intervention was carried out under medical supervision and all the participants continued with their routine medical treatment, complying with the beneficence and non-maleficence principles of bioethics [47].

### 2.5. Statistical Analysis

The obtained data were analyzed using Jamovi 1.8.4. After completing the descriptive analysis of the variables, which is shown in Table 1 for the numerical variables (mean ± SD) and in the table for categorical variables (%), the homogeneity of ages between both groups was contrasted by means of the Student’s test.

The differences between groups in terms of drug intake were evaluated, in the pre-intervention period and during the intervention, by means of the χ^2^ test (Table 2). Moreover, the changes between the two phases of drug consumption were evaluated for both groups using McNemar’s test (Table 2).

The normality of each of the six main outcome measures (distinguishing between pre- and post-intervention) was analyzed using the Schapiro–Wilk test, where both experimental groups were considered separately. Considering that 18 of the comparisons were non-significant, a repeated-measures model (with Greenhouse–Geisser’s correction) was selected and applied to each of the six measures, with pre–post as the intra-subject factor and two inter-subject factors, with the experimental group (control–wine) as the main one and, as the secondary one, the consumption of the type of drug. This second factor was included with the aim of controlling a potential confounding variable due to the results obtained in Table 2. More specifically, in the analysis of pain, whether or not the participant had used analgesics during the intervention was controlled for; for the assessment of tender points, the intake of anti-inflammatory drugs was controlled for; for sadness, we controlled for selective serotonin reuptake inhibitors (SSRIs); for anxiety, we controlled for benzodiazepines; and for depression, we controlled for tricyclic antidepressants. In the case of FIQ, no specific consumption was considered.

To assess the effect of wine consumption (Table 1), we mainly focused on the result of the interaction between the intra-subject factor (pre–post) and the main inter-subject factor (control–wine), as well as the results of the post hoc comparisons between groups, in both the pre-intervention and post-intervention periods. As a secondary target, the differences between before and after in both groups were also analyzed. Post hoc comparisons between estimated marginal means were carried out according to Fisher’s Least Square Difference (LSD).

The study relied upon the availability of the members of the Fibromyalgia Associations to participate. Therefore, the sample size was not based on a prior power calculation, but instead on the largest possible number of women who met the selection criteria. The sample size that we finally obtained allows us to detect an effect size of Cohen’s d = 0.845 for a significance level of α = 0.05 and a power of 0.80.

## 3. Results

**Figure 1:**
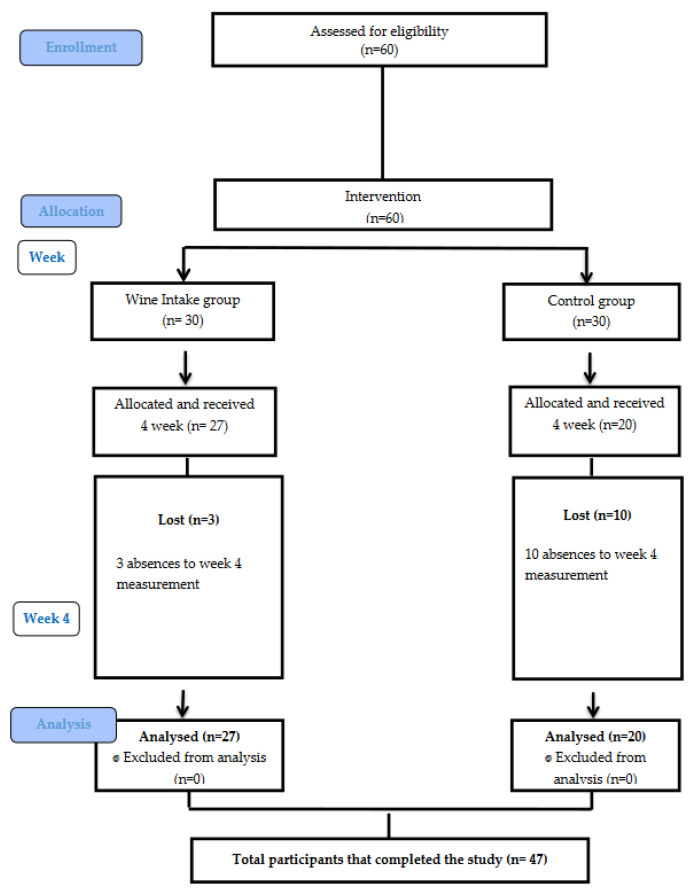
CONSORT flow chart.

A total of 47 participants completed the study: 20 in the control group and 27 in the experimental group. A CONSORT flow diagram is given in Figure 1. None of the experimental group’s participants reported side effects for the light intake of the red wine administered. The mean age of the patients was 46.87 ± 9.97 years, specifically, 49.35 ± 0.66 for the control group and 45.04 ± 8.28. There was no significant difference between the groups (*p* = 0.145). They had a mean number of years since diagnosis of the disease of 5.34.

At the beginning of the study, all the participants were taking some kind of drug. It should be noted (Table 2) that the criteria for assignment to the control and experimental groups were strongly related to prior consumption of SSRIs (*p* = 0.002), benzodiazepines (*p* < 0.001), and tricyclic antidepressants (*p* = 0.020). The case of benzodiazepines was particularly notable, given that none of the participants in the experimental group consumed them during the period of intervention. In addition, since the percentages of consumption hardly varied during the weeks of the intervention (see McNemar’s test results in Table 2), it was necessary to include this consumption as an inter-subject factor in each repeated-measures model proposed, as mentioned in the previous section.

As a result of the repeated-measures model, we point out first that, according to post hoc comparisons (Table 1), both groups could be considered homogenous in the six scales considered before treatment.

**Figure 2:**
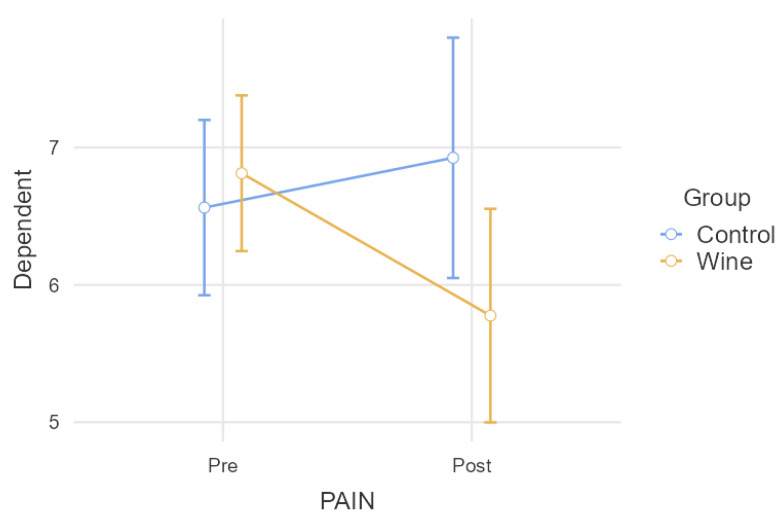
Comparative analysis of pain when controlling the intake of analgesic medication.

When performing the comparative analysis of pain, controlled according to the intake of analgesic medication (Table 1), the model showed a significant result (*p* = 0.016) in the interaction between the group and the intra-subject factor, i.e., the groups changed in terms of the mean in significantly different ways (as shown in Figure 2). Differences between groups after the intervention were very close to significance (*p* = 0.054). Additionally, there was a significant decrease in pain in the wine group (*p* = 0.008), while the control group did not change significantly (*p* = 0.390). This is why the interaction is so clear in this case.

**Figure 3:**
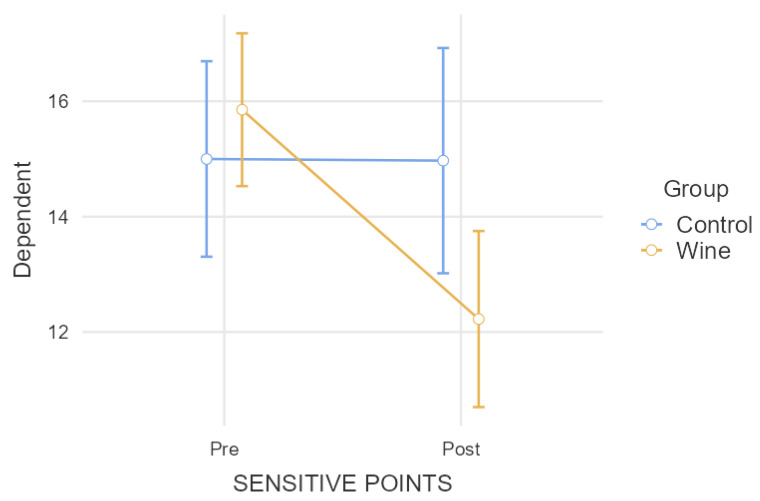
Comparative analysis of tender/sensitive points when controlling the drug intake.

In the comparative analysis of the number of controlled tender points according to the consumption of anti-inflammatory drugs (Figure 3), the repeated-measures model showed, according to Table 1, a significant result (*p* = 0.007) in the interaction between the group and the intra-subject factor. A comparison between the groups after the intervention showed a significant difference (*p* = 0.031). Moreover, a decrease in pain in the experimental group (*p* < 0.001) was found, while the control group did not change (*p* = 0.977).

**Figure 4:**
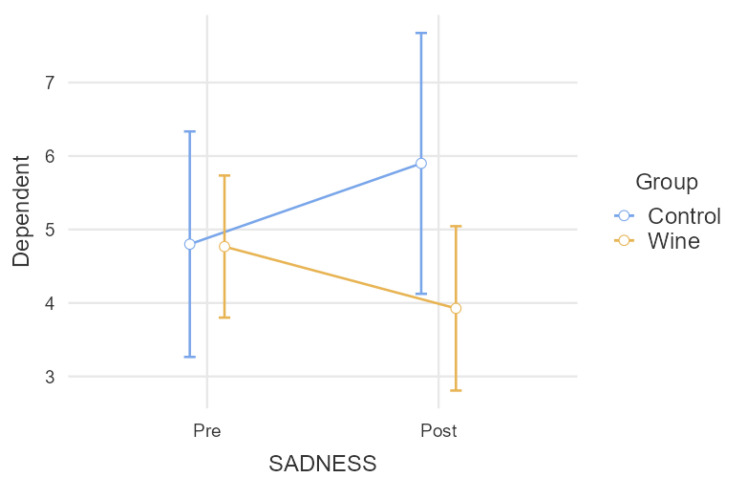
Comparative analysis of sadness when controlling the SSRI intake.

Regarding the comparative analysis of sadness, controlled according to SSRI consumption (Figure 4), the repeated measures model showed a significant result (*p* = 0.018) in the interaction between the group and the intra-subject factor. As seen in Table 1, the difference between both groups was close to significance post-intervention (*p* = 0.065), as was the pre–post difference for the wine group (*p* = 0.52).

**Figure 5:**
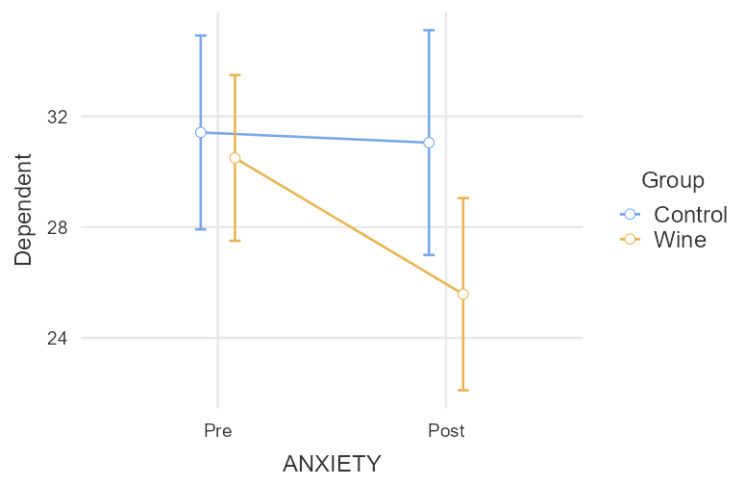
Comparative analysis of anxiety levels.

In the comparative analysis of anxiety (Figure 5), benzodiazepine intake could not be included in the repeated measures model due to the low consumption of this medication in the experimental group. In any case, the result was close to significance (*p* = 0.078) in terms of the interaction and a significant difference was found between groups after the intervention (*p* = 0.038). Additionally, we found an intra-subject significant decrease (*p* = 0.028) for the experimental group, while the control group did not change (*p* = 0.957).

For the comparative analysis of depression, controlled according to tricyclic drug consumption, the repeated-measures model showed a non-significant result (*p* = 0.737) in the interaction between the group and the intra-subject factor. No significant results were obtained in the post hoc comparisons (see Table 1).

The last comparative analysis performed was for the FIQ, in which the repeated-measures model showed a non-significant result (*p* = 0.202) in the interaction between the group and the intra-subject factor. In addition, no significant results were obtained in the post hoc comparisons, although the difference between the groups was very close to significance (*p* = 0.058).

## 4. Discussion

Spain is a Mediterranean country where, according to tradition, wine is a beverage that is part of the regular diet. For this reason, the objective of this pilot study was to analyze whether the light consumption of wine could help to improve the main symptoms in women with fibromyalgia. We obtained significantly favorable results in relation to pain, tender points, and anxiety and noted a clear improvement in the evolution of sadness, depression, and quality of life. Fibromyalgia has no curative or specific treatment, which leads to polypharmacy for patients with this condition. Therefore, we considered it essential to include drug consumption as a possible confounding factor in the statistical model. The results were not significant, although they were significant when analyzing the wine–evolution interaction. This suggests that the differences in the evolution of the two groups (experimental and control) should not be attributed to the consumption of these drugs, but to the consumption or non-consumption of wine.

The scientific evidence demonstrates that, in fibromyalgia, there is a decrease in the levels of serotonin and that this substance can then be increased with red wine consumption [32,33]. Fibromyalgia has been widely reported on in the medical literature as a disease that is increasing in incidence, and approaches to treating fibromyalgia are multi-factorial, with highly diverse results [3,4,7,8,9]. Concerning wine, we also found evidence in multiple medical studies that demonstrated the effectiveness of its moderate consumption on the prevention of relevant diseases with a high grade of prevalence and mortality [12,13,14,17,22,23,27,29,30,31].

Our results showed that both pain and tender points decreased in a statistically significant way in the experimental group, suggesting that this decrease was not related to the consumption of anti-inflammatory drugs or analgesics, respectively. In this way, the pain relief obtained in our study with the light intake of red wine coincides with the results of Panconesi [48], which showed how low doses of alcohol can have a beneficial effect in patients with migraines.

Moreover, studies conducted in animals indicate that wine consumption improves depression [49] and anxiety [50]. In our study, the patients in the experimental group showed significant improvements in levels of anxiety. A study carried out by Nickel et al. [51] concluded that 69% of patients that present medically unexplainable pain syndromes suffer from anxiety and depressive disorders. They also found that the prevalence of fibromyalgia increased with the grade of somatization that the person experienced. We do not agree with these data as, in our study, anxiety was decreased in the experimental group, and we contend that this effect was mostly likely not only psychological but also organic.

It is important to mention that, due to the low consumption of benzodiazepines by the women in the experimental group, it was not possible to introduce this drug intake into the repeated-measures model in the comparative analysis of anxiety. However, it resulted in a statistically significant improvement in the anxiety levels of this group, while these levels were maintained in the control group.

Along these same lines, sadness (evaluated with the VAS) also showed a very clear and significant improvement in the experimental group. ‘Sorrows with wine are less sorrows’ is a saying that is deeply rooted in popular Mediterranean culture. Due to the fact that this truism is deeply rooted in our culture, we must also discuss the possibility of a placebo effect regarding the consumption of wine and the improvement of wellbeing. However, a placebo group was not included in our study due to the lack of patients, which made it impossible to create a third study group. On the other hand, a study carried out by Guallar et al. [52] at the Central University of Public Health, Autonomous University of Madrid (Spain), on the consumption of alcoholic beverages and subjective health in Spain concluded that moderate drinkers (while the consumption of wine and beer was higher) had a less pronounced perception of better subjective health.

Along with the improvements in the symptoms of pain, tender points, and anxiety, we found that, although the post hoc comparative results were not significant for the level of sadness, depression, and quality of life, the observed mean values indicated a better evolution of the women in the experimental group.

The patients in the experimental group showed a decrease in the average levels of depression (from major to light), which correlates with the findings of Smith et al. [53], who demonstrated that the administration of beverages containing tryptophan improves the symptoms of depression.

In relation to the impact of fibromyalgia, we think that the improvements obtained in the rest of the outcome measures could have influenced the results of the fibromyalgia impact questionnaire. The total score of the FIQ was also significantly improved in the experimental group. This is important even though the decrease was not found to be a result of the seven points that authors such as Bennett [54] considered inflexible in order to assess the effectiveness of a treatment. In this respect, we should highlight that, at the beginning of the study, our patients had average levels of fibromyalgia impact that are considered to be at the minor severity limits. In other words, the FIQ was less than 70 and the experimental group’s score still decreased by an average of 3.5 points, reflecting a significant improvement in the quality of life of the patients in this group. In contrast, this did not occur in the control group.

In addition, age (with an average age of 46 years old, which coincides with the most frequent mean age for women suffering from fibromyalgia [4,6,7]), along with the mean time from fibromyalgia diagnosis (5 years for chronic characteristics of the disease) [4,6,7], did not influence the results of this study.

Regarding the type of wine administered, Tempranillo red wine was chosen because of its lower sugar content and higher polyphenol content. It would be interesting in future studies to see whether there are differences between different types of wine and, in particular, to add a placebo group of must intake, as it lacks alcohol content.

Finally, it is important to discuss the controversy related to wine consumption in relation to the presence of alcohol. Wine contains many of the beneficial components that grapes have, such as tannins, which have antioxidant effects, or resveratrol, with its positive health effects. However, wine also has a component that is formed during the fermentation of the must, and that is alcohol, which has both positive and negative health effects [55]. The presence of alcohol in wine means that there is a great deal of controversy about the effects of these components on the body, and whether these alcohol-containing beverages are harmful or, on the contrary, beneficial to health. To date, there is no consensus as to whether the benefits or negative effects are not based on a unilateral relationship, in which only wine and the health of the person are involved. Instead, there are other factors involved, such as the patient’s age, gender, health status prior to the study, socioeconomic status, level of involvement, and knowledge about the world of wine, among many others [56].

It has been proven by numerous studies that the non-alcoholic compounds in wine have protective and positive effects against some diseases such as cardiovascular problems, blood sugar, lower blood cholesterol (LDH), increased antioxidant capacity, some types of cancer, and bone problems such as osteoporosis, among others [55]. It is also known that alcohol has negative effects both in the short term, such as aggressive and violent behaviours, increased suicidal tendencies, etc., and in the long term, such as in cirrhosis of the liver, pancreatitis, certain types of cancer, especially some types of breast and stomach cancer, stroke, and foetal alcohol syndrome [57]. These negative effects are associated with excessive alcohol consumption, and, in fact, alcohol is the third leading cause of death in the world [56].

In recent years, research into the possible positive effects of alcohol on the body has been conducted, with many studies concluding that alcohol has protective and reducing effects on cardiovascular disease [58] due to an increase in HDL and a decrease in LDL, as well as a decrease in platelet aggregation [59,60]. In addition, decreases in the rates of diabetes-associated problems [61] or problems related to heart attacks [62,63] have been observed. The most important aspect to bear in mind is that the beneficial effects of alcohol only occur at low doses, with moderate consumption, and increase when consumed with food; these factors were considered in the intervention made in this study.

### Study Limitations

Considering that the sample size should be obtained from a target population that is already limited to the small number of members of the two participating associations (AFIBA, AFIBROL) and that the intervention factor to be studied, red wine, involved alcohol consumption (although always within the limits set by the WHO as low-risk light consumption), we decided not to carry out a randomized controlled trial. That is the reason why we chose to conduct a longitudinal before–after pilot study with a control group.

The distribution of the sample cannot be left to chance (the principle of beneficence) [47]. Furthermore, to avoid assuming excessive risks (the principle of non-malfeasance) [47], we tried to avoid bias in the distribution of the groups (based on the inclusion and exclusion criteria established). This was achieved by providing a homogeneous sample for the experimental and control groups, which were treated in a similar manner throughout the study, except for the wine intake. This is shown in the statistical analysis and thus ensures internal validity. Given the peculiar characteristics of our study factor, wine as a food with fermented alcohol content was administered to patients on medication, and this fact, with maximum rigor applied, would have made it impossible to carry out the study, given that the vast majority of drugs should not be consumed together with drugs. Hence the impossibility ensuring the distribution of the groups, because, assuming the bias, we considered it appropriate to decide, under medical control, which patients could not be part of the intervention group due to the type and quantity of drug consumed. Both of these factors influenced the small sample size. Moreover, due to this initial heterogeneity, we were more interested in the evolution of the experimental group than in differences between the two groups.

This is a preliminary study that was therefore carried out with a small sample size. This increases the probabilities of type II errors, especially if we had applied Bonferroni’s corrections. Although there were fewer than 50 participants, the fact that the statistical results are significant, particularly for sensitive points (even with Bonferroni’s correction), encourages us to continue in this line of research by increasing the sample size. The losses experienced during the study were expected because fibromyalgia patients are frequently motivated at the beginning of the treatments, but this motivation decreases over time [3,4,6,7,8]. Nevertheless, the sample size proved to be statistically sufficient.

For the data collection, information and memory biases were avoided by using the structured questionnaire for the quantitative measurement of the variables, trusting in the sincerity of the responses and in the participants’ agreement to the stipulations.

As diet is a confounding factor to be taken into account; a diary was prepared so that each of the participants could record their intake of both solid and liquid foods, as well as the approximate amount eaten. These diaries were not analyzed in the results of the study, because, at the end of the intervention, less than 5% of the participants had completed it properly, so it was decided to assume the risk arising from the lack of control of the diet, given that the food (products and consumption habits) in the area of residence of all participants (the city of Badajoz) was very similar, if not practically the same, as the Mediterranean diet is predominant in the area. It would also have been useful to collect additional information on the sample’s alcohol consumption prior to enrolment in the study.

These results should be considered preliminary due to the small simple size. Therefore, we will continue along this line of research, increasing the sample size, including a placebo group, and performing serological measurements of serotonin, which could not be carried out in the present study due to lack of financial support. These further studies will show whether the level of this neurotransmitter, which is deficient in women with fibromyalgia, really increases with the light intake of red wine, or whether this result could be due to the placebo effect and whether this increase is the cause of the improvement achieved in the intervention group.

## 5. Conclusions

Based on the results of the present pilot study, the light consumption of alcohol in the form of red wine as part of the diet and under medical supervision improved the main symptoms of fibromyalgia (pain, tender points, anxiety, sadness, and depression) and reduced the impact of the disease on the women studied. However, future studies with a bigger sample size and a controlled diet are necessary to confirm these preliminary data. It would also be interesting to analyze the mechanisms through which improvements are achieved.

## Figures and Tables

**Table 1:** Baseline and post-intervention outcomes for the control and experimental groups, the difference between both groups, and the interactions between the pre–post and control groups.

		Control (N = 20)	Wine (N = 27)	Inter-Group Difference	Interaction
A: Pain (0–10)	VAS before	6.50 ± 1.54	6.89 ± 1.22	*p* = 0.557 ^b^	*p* = 0.016 ^a^
	VAS after	6.89 ± 1.49	5.89 ± 1.99	*p* = 0.054 ^b^	
	Intra-group difference	*p* = 0.390 ^c^	*p* = 0.008 ^c^	--	
B: Sensitive points (0–18)	# before	14.83 ± 3.43	15.85 ± 3.23	*p* = 0.427 ^b^	*p* = 0.007 ^a^
	# after	14.76 ± 3.51	12.22 ± 4.12	*p* = 0.031 ^b^	
	Intra-group difference	*p* = 0.977 ^c^	*p* < 0.001 ^c^	--	
C: Sadness (0–10)	VAS before	5.56 ± 2.48	4.63 ± 2.42	*p* = 0.971 ^b^	*p* = 0.018 ^a^
	VAS after	5.61 ± 2.73	3.78 ± 2.78	*p* = 0.065 ^b^	
	Intra-group difference	*p* = 0.106 ^c^	*p* = 0.052 ^c^	--	
D: Anxiety (0–56)	HAS before	31.42 ± 8.05	30.59 ± 7.08	*p* = 0.672 ^b^	*p* = 0.078 ^a^
	HAS after	31.05 ± 9.16	25.58 ± 8.49	*p* = 0.038 ^b^	
	Intra-group difference	*p* = 0.957 ^c^	*p* = 0.006 ^c^	--	
E: Depression (0–52)	HDRS before	18.95 ± 4.42	18.63 ± 5.08	*p* = 0.144 ^b^	*p* = 0.737 ^a^
	HDRS after	20.17 ± 4.38	16.62 ± 4.50	*p* = 0.166 ^b^	
	Intra-group difference	*p* = 0.775 ^c^	*p* = 0.112 ^c^		
FM impact (0–100)	FIQ before	60.84 ± 16.80	59.56 ± 14.10	*p* = 0.851 ^b^	*p* = 0.202 ^a^
	FIQ after	60.76 ± 12.72	53.48 ± 11.62	*p* = 0.058 ^b^	
	Intra-group difference	*p* = 0.939 ^c^	*p* = 0.053 ^c^		

NOTE: VAS: Visual Analogue Scale; FM: fibromyalgia; SSRI: selective serotonin reuptake inhibitors; HAS: Hamilton Anxiety Scale; HDRS: Hamilton Depression Rating Scale. ^a^ Interaction between the intra-subject factor and the main inter-subject factor from a repeated measures model, with pre–post as the intra-subject factor, control–wine as the main inter-subject factor, and, as a secondary inter-subject factor (except for the anxiety and FM analyses), the intake of the potentially most confounding medication. ^b^ LSD post hoc comparisons between the estimated marginal means of the main inter-subject factor groups (wine–control). ^c^ LSD post hoc comparison between estimated marginal means of the intra-subject factor (pre–post).

**Table 2:** Medication intake before and during the intervention.

		Control (N = 20)	Wine (N = 27)	Inter-Group Difference
A: Analgesic	% before	55.0	77.8	*p* = 0.098 ^b^
	% follow-up	60.0	70.4	*p* = 0.458 ^b^
	Intra-group difference	*p* = 0.317 ^a^	*p* = 0.317 ^a^	--
B: Anti-inflammatory	% before	65.0	55.6	*p* = 0.514 ^b^
	% follow-up	65.0	51.9	*p* = 0.367 ^b^
	Intra-group difference	*p* = 1 ^a^	*p* = 0.705 ^a^	--
C: SSRI	% before	85.0	40.7	*p* = 0.002 ^b^
	% follow-up	85.0	37.0	*p* = 0.001 ^b^
	Intra-group difference	*p* = 1 ^a^	*p* = 0.317 ^a^	--
D: Benzodiazepine	% before	55.0	3.7	*p* < 0.001 ^b^
	% follow-up	50.0	0	*p* < 0.001 ^b^
	Intra-group difference	*p* = 0.317 ^a^	--	--
E: Tricylic antidepressant	% before	10.0	40.7	*p* = 0.020 ^b^
	% follow-up	5.0	37.0	*p* = 0.010 ^b^
	Intra-group difference	*p* = 0.317 ^a^	*p* = 0.564 ^a^	--

^a^ McNemar test to compare pre–post for each group. ^b^ χ^2^ test to compare groups for each phase.

## Data Availability

The data underlying this article cannot be shared publicly to maintain the privacy of the individuals who participated in the study. The data will be shared on reasonable request to the corresponding author.
